# Comparing Associations of Dietary Energy Density and Energy Intake, Macronutrients with Obesity in Patients with Type 2 Diabetes (JDDM 63)

**DOI:** 10.3390/nu13093167

**Published:** 2021-09-11

**Authors:** Yasunaga Takeda, Kazuya Fujihara, Rina Nedachi, Izumi Ikeda, Sakiko Yoshizawa Morikawa, Mariko Hatta, Chika Horikawa, Mitsutoshi Kato, Noriko Kato, Hiroki Yokoyama, Yoshio Kurihara, Kazuhiro Miyazawa, Hiroshi Maegawa, Hirohito Sone

**Affiliations:** 1 Department of Internal Medicine, Faculty of Medicine, Niigata University, Niigata 951-8510, Japan; mr2.yac@gmail.com (Y.T.); rina.nedachi.t@gmail.com (R.N.); ikdizm12@gmail.com (I.I.); marichocolate.coffee02@gmail.com (M.H.); sone@med.niigata-u.ac.jp (H.S.); 2 Niigata University Medical & Dental Hospital, Niigata 951-8520, Japan; 3 Department of Food Science and Dietetics, Faculty of Human Life Science, Tokushima Bunri University, Tokushima 770-8514, Japan; sakiko.1211@gmail.com; 4 Department of Health and Nutrition, Faculty of Human Life Studies, University of Niigata Prefecture, Niigata 950-8680, Japan; horikawa@unii.ac.jp; 5 Kato Clinic of Internal Medicine, Tokyo 125-0054, Japan; katom@gol.com (M.K.); norikokato.0131@gmail.com (N.K.); 6 Jiyugaoka Medical Clinic, Obihiro 080-0016, Japan; dryokoyama@yokoyamanaika.com; 7 Kurihara Clinic, Sapporo 004-0053, Japan; ykuri@yg7.so-net.ne.jp; 8 Miyazawa Clinic, Kofu 081-0201, Japan; miyakazu@crest.ocn.ne.jp; 9 Department of Medicine, Shiga University of Medical Science, Otsu City 520-2192, Japan; maegawa@belle.shiga-med.ac.jp

**Keywords:** energy density, energy intake, obesity, type 2 diabetes mellitus

## Abstract

To investigate the association between dietary energy density (DED) and obesity in people with type 2 diabetes mellitus. Moreover, we compared the strength of the associations of DED with intake of energy and macronutrients in terms of obesity as well as nutritional factors that have long been used for medical nutritional therapy. Cross-sectionally investigated were 1615 outpatients with type 2 diabetes who attended 26 clinics nationwide with diabetes specialists. Odds ratios (ORs) were calculated for the association between obesity and DED, energy, and macronutrients by quintile categories and a 1 SD increment with adjustment for potential confounders. β coefficients were calculated for the association between body mass index (BMI) and each nutritional factor by 1 SD increments in nutritional values. Multi-adjusted OR for obesity between extreme quintiles of DED was 2.99 (95% confidence interval (95% CI): 2.01–3.12). Conversely, the ORs did not differ significantly according to the quintiles of other nutrient factors. Multi-adjusted β coefficient of BMI per 1 SD according to DED was far higher than those of other nutrient factors (β coefficient 0.65, 95% CI: 0.41–0.88). These findings indicated that DED in persons with type 2 diabetes was positively associated with BMI and the prevalence of obesity. DED was also much more potently associated with obesity and BMI than nutritional indicators such as intake of energy or macronutrients.

## 1. Introduction

Obesity is strongly associated with the development of type 2 diabetes mellitus and its complications [1,2,3,4,5]. In a randomized controlled study of overweight or obese patients with type 2 diabetes, weight loss was shown to lead to improved insulin response and diabetes remission [6]. Lifestyle modifications such as diet and/or exercise are recommended in most therapeutic guidelines for the fundamental care of people with type 2 diabetes [7,8,9]. Guidelines for diabetes also recommend diet therapy to prevent the onset and progression of diabetic complications [10]. Moreover, an intervention study for patients with type 2 diabetes in Japan reported that lifestyle modification, including diet, significantly reduces the risk of stroke incidence [11]. Especially, diet therapy plays a crucial role in the management of obesity in type 2 diabetes, with the restriction of energy intake being the primary approach [7,8,9]. Low-fat or low-carbohydrate diets also have been shown to have an effect on weight loss [12,13]. However, all of these conventional diet therapies were known to reduce patient satisfaction, making their continuation for extended periods difficult.

Dietary Energy density (DED), that is, the amount of energy per unit weight of food, has recently attracted attention as an indicator reflecting the quality of the overall diet [14]. Foods can be divided into four categories, based on the their DED (very-low: less than 0.6 kcal/g, low: 0.6 to 1.5 kcal/g, medium: 1.5 to 4.0 kcal/g, high: more than 4.0 kcal/g) [15,16]. A diet with high DED is rich in fat and added sugar, and lower water content, such as snacks and cookies [17]. Conversely, low DED diets are rich in dietary fiber and different vitamins and water, such as in vegetables and fresh fruits [18]. Cross-sectional studies in the general population showed that high DED is positively associated with the prevalence of obesity and metabolic syndrome [18,19,20]. Longitudinal studies of the general population in Western countries showed that higher DED at baseline contributes to not only weight gain [21] but also to obese-related cancers [22]. However, the components of a meal are different from those in Japan. The Japanese dietary pattern characterized by high water content and higher intakes of white rice, legumes, fish, vegetables, and lower intakes of fats, oils, and soft drinks compared to Western diets were low-density diets [23]. Previous interventional studies in people who were overweight or obese reported that the traditional Japanese diet contributed to improving anthropometric indices, HbA1c, HDL-C, LDL-C [24,25]. Therefore, Japanese diets have been of interest worldwide because they may prevent the onset and progression of coronary heart disease and type 2 DM.

However, to our knowledge, no study has examined the association between DED and obesity in type 2 diabetes. Moreover, regardless of the presence or absence of diabetes, we are not aware of studies that have compared DED with other nutritional factors that have been strongly associated with obesity (i.e., total energy, amount of macronutrients). Therefore, the aim of this study was to clarify the association between obesity and DED among people with type 2 diabetes and to compare the degree of the strength of associations with other nutritional factors that have long been used for medical nutritional therapy. Findings of these studies could be used for effective as well as easy-to-understand dietary education and for dietary interventions with lower reductions in quality of life for obese people with type 2 diabetes.

## 2. Methods and Materials

### 2.1. Study Participants

Participants in this cross-sectional study were outpatients with type 2 diabetes treated at 26 clinics participating in the Japan Diabetes Clinical Data Management Study Group (JDDM). We analyzed data on 2052 patients with type 2 diabetes from 26 clinics that distributed lifestyle questionnaires from the JDDM from 1 December 2014 to 30 October 2017. Among them, 1984 patients completed the dietary survey. Furthermore, those with defects and outliners in the anthropometry measurements and clinical data and prescription of oral hypoglycemic agents (OHA) were excluded. (*n* = 369) The final sample included 1615 patients.

### 2.2. Outcome Measure

Height and weight data were obtained by a self-administered questionnaire. Participants were instructed to fill out a questionnaire with their recent height and weight. Body mass index (BMI) was calculated as weight in kilograms divided by height in meters squared and rounded to the nearest tenth. Obesity was defined as a BMI of 25.0 or higher [26].

### 2.3. Dietary Assessment

A dietary survey was conducted using a food frequency questionnaire based on food groups (FFQg). The FFQg consists of total 34 questions and elicits information on the average intake per week of 29 food groups and 10 kinds of cookery in commonly used units or portion sizes. The FFQg was externally validated by comparison with weighed dietary records for 7 continuous days of 66 study participants aged 19–60 years [27]. We used standardized software for population-based surveys and nutrition counseling in Japan (EIYO-KUN v6.0, developed by Shikoku University Nutrition Database; KENPAKUSHA, Tokyo, Japan) to calculate nutrient and food group intake.

### 2.4. Calculation of DED

Energy density was calculated by dividing total energy intake by the amount of edible food. Methods for calculating DED based on the inclusion of beverages were associated with higher variance ratios, which may diminish associations when examining health outcomes [28]. Therefore, in this study, caloric and non-caloric beverages were excluded from calculations.

### 2.5. Other Variables

Clinical data and prescription of OHA and insulin were extracted by software from the JDDM from 1 December 2014 to 30 October 2017. Blood pressure and biochemical values (HbA1c, lipid metabolism factors) were measured at each participating clinic or hospital. Non-HDL cholesterol was calculated by subtracting the HDL cholesterol value from the total cholesterol value. The amount of physical activity was calculated using the Japanese version of the International Physical Activity Questionnaire (IPAQ) short form [29]. Smoking status was obtained from a questionnaire.

### 2.6. Statistical Analysis

The characteristics of JDDM participants were described as mean ± SD and percentages. Differences in characteristics and nutritional intake by quintiles of DED tested were determined by ANOVA for continuous variables and by χ2 tests for categorical variables.

Linear regression analysis was used to calculate the β coefficient and 95% CI for the association between BMI and DED, energy, and macronutrients by each 1 SD increment in nutritional values. Logistic regression analysis was used to calculate the odds ratio (OR) and 95% CI for the association between obesity and each nutritional value by 1 SD increments in nutritional values and quintile categories in comparison with the first quintile adjusted for sex, age, diabetes duration, treated by insulin, treatment by OHAs, physical activity, smoking status, and alcohol intake in the analyses. Linear regression analysis was used to calculate the standardized coefficient to identify food groups correlating with DED.

All statistical analyses were performed by SPSS (version 19.0, Chicago, IL, USA), and statistical significance was considered for *p* < 0.05.

## 3. Results

Baseline characteristics and nutritional and food group intakes of the 1615 study participants stratified by quintiles of DED are shown in Table 1. The mean value and SD of DED were 1.53 and 0.23, respectively, in the participants overall. The mean DED value in the highest quintile of DED was approximately 1.5 times higher than that in the lowest quintile. There were significant trends in differences in BMI among quintiles, with the BMI in the highest DED quintile approximately 14% higher than that in the lowest quintile. Blood pressure, non-HDL cholesterol, and medications for diabetes did not differ significantly according to quintiles.

In terms of food groups, higher quintiles of DED were associated with significantly greater intakes of bread, meats, confectioneries, and beverages other than alcohol and significantly smaller intakes of brightly colored and other vegetables, seaweed, beans, milk/dairy products, fruits, and sugar.

To compare the impact of each nutrient factor on the prevalence of obesity, we calculated the ORs of having obesity (i.e., BMI ≥ 25) according to each quintile of DED and other nutrient factors that are known to be associated with obesity estimated by logistic regression models adjusted for age, sex, and multiple variants (diabetes duration, treated by insulin, treated by OHA, physical activity, current smoking, alcohol intake) (Table 2). In confounder-adjusted logistic regression, the prevalence of obesity differed significantly from the first to the fifth quintiles of DED; the OR between extreme quintiles was almost 3.0. On the other hand, the prevalence of obesity did not differ significantly according to the quintiles of other nutrient factors.

To further confirm the prominent impact of DED on obesity, we compared ORs and multivariate β coefficients per SD increment of each nutrient factor on the prevalence of obesity (Table 3) as well as BMI (Table 4), respectively. In both age and sex-adjusted models and multivariate-adjusted models, both the ORs and β coefficients of DED were demonstrated to be far higher than those of other nutrient factors, including energy and fat, although energy and fat were also shown to be significantly associated with BMI and obesity.

ORs for obesity with 95% CIs for 1 SD increase of DED, energy, macronutrients analyzed by logistic regression analysis. Logistic regression models adjusted for age, sex, diabetes duration, treated by insulin, treated by OHA, physical activity, smoking status, alcohol intake.

ORs for obesity with 95% CIs for DED, energy, macronutrients according to quintiles (ORs for the lowest quintile as reference are shown) analyzed by logistic regression analysis. Logistic regression models adjusted for age, sex, diabetes duration, treatment by insulin, treatment by oral hypoglycemia agents, physical activity, smoking status, and alcohol intake

Based on the strong association between DED and the prevalence of obesity shown in our previous analyses, we further identified food groups that were positively or negatively correlated with DED (Table 5). Standardized β coefficients of DED in each food group by multivariate linear models demonstrated that confectioneries, bread, fat and oil, meat, and seeds were significantly positively associated with DED. On the contrary, eggs, noodles, beans, dairy products, potatoes, fish and seafood, steamed white rice, green and yellow vegetables, fruits, and vegetables other than green and yellow vegetables were negatively associated with DED. The top five food groups that showed a particularly strong negative association with DED were vegetables not classified as green or yellow, fruits, green and yellow vegetables, steamed white rice, fish and seafood.

## 4. Discussion

Our results revealed that a diet with high DED was positively and prominently associated with the prevalence of obesity independently of confounding factors, including physical activity or anti-diabetic drug therapy in people with type 2 diabetes, as was previously shown in individuals without diabetes [19,20]. Moreover, we also found that the association of DED with obesity was far stronger than that of other typical nutrient factors that have been established to be associated with obesity, such as fat or carbohydrate.

Mean DED of our participants of 1.53 kcal/g was considerably lower than that reported in previous studies in Western countries, which ranged from 1.70–1.99 kcal/g [18,30,31]. The Western diet, in general, was reported to load mainly high energy-dense foods [32]. This relatively lower DED seen in the Japanese diet might have resulted from higher consumption of foods with a high water content (such as steamed white rice, noodles, vegetables, seafood and fish) and lower consumption of high-dense foods (such as oil and fat, confectioneries) than in Western countries. Findings similar to the present study were also obtained in general populations in Japan [33].

Our study in people with type 2 diabetes showed positive associations between DED and obesity or BMI. These associations were similarly seen in previous cross-sectional studies in general Western populations [18,19]. If the amount of energy is the same for two particular foods, the portion to be consumed of the food with the higher DED should be expected to be significantly smaller than the food with a lower DED [19]. However, since it was shown that people habitually consume a diet that is fairly consistent regarding the actual weight of the food, the consumption of foods with high DED that contain more energy per gram might increase overall energy intake [34]. Thus, a habitual diet of foods with high DED could be associated with higher BMI or a higher prevalence of obesity. Additionally, we found that the OR for obesity in patients in the 4th quintile of DED (≥1.71 kcal/g) was much higher than in the 3rd or lower quartiles, which suggested that DED of 1.7 could be a threshold for weight management in Japanese with type 2 diabetes, although an interventional study is needed to prove this.

We firstly found by linear and logistic regression analyses that DED was strikingly and more potently associated with BMI and the prevalence of obesity than the intake of energy or macronutrients. This suggests that a diet with DED contains more factors that are closely related to increases in BMI or the development of obesity compared to the amount of energy or macronutrients alone, regardless of the volume of food. One such factor is speculated to be the amount of dietary fiber. In this study, the low DED diet was closely associated with increased vegetable and fruit intake, which was almost parallel to the amount of dietary fiber. Dietary fiber is known to reduce the overall absorption of energy-providing nutrients such as fat and protein [35]. Thus, diets rich in fiber could contribute to the prevention of obesity. Another such factor is considered to be food groups. Previous studies reported that high DED diets such as the Western diet contain a lot of meat, confectioneries, fats, and oils. In this study in Japan, DED was also positively associated with such food groups. Meat is rich in animal fat, and confectioneries are rich in added sugars, which reportedly contribute to weight gain [32,36]. In addition, recent studies reported that these foods could increase the amount of energy absorption through changing bacterial flora in the intestine and affect weight gain [37].

As a whole, DED is associated with diet quality rather than individual nutritional factors such as energy and macronutrients, which could explain the stronger association of DED with weight status rather than other established nutritional elements.

The current study has several strengths. First, to the best of our knowledge, this is the first study to investigate the association between obesity and DED in patients with type 2 diabetes. This is also the first study regardless of the presence or absence of diabetes to compare the strength of the association between obesity and DED, energy intake, and macronutrient intake such as fat and carbohydrates. Three approaches were used to examine the relationship between each nutritional factor and BMI and obesity in a large number of patients with diabetes. Briefly, the odds ratios for the association between obesity and various nutritional factors according to quintiles were determined, β coefficients for the associations between BMI and per 1 SD increment of such nutritional factors were analyzed, and finally, odds ratios for the association between obesity and per 1 SD increment of such nutritional factors were examined. We revealed that DED had the strongest positive association with BMI and obesity compared with other nutritional factors or indices. Another strength is the nationwide sampling of patients. These findings will enable the generalizability of our results in relation to common patients with type 2 diabetes in Japan. Several limitations of the present study must be considered. First, this study was cross-sectional and, therefore, unable to unravel the causal relationships between exposure and outcome. There have been longitudinal and interventional studies that examined the association between DED and BMI and obesity [21,38]. These studies showed that DED was positively associated with weight gain and higher BMI over time, and reduction in DED was associated with weight loss in the general population with overweight or obesity. Therefore, further longitudinal and interventional studies are also needed to examine the associations among patients with diabetes. Second, that participants reported inaccuracies regarding their dietary composition on the self-recording questionnaire is possible [39]. Additionally, since the DED value is strongly dependent on fat and water content, methods of cooking can affect these values. However, in this study, we could not evaluate DED according to cooking methods, as the food frequency questionnaire that we used did not elicit this information. Finally, although the intake of energy among patients with diabetes in Western countries is not much different from that in Japanese patients, patients in Western countries had higher BMIs than Japanese patients. It can be considered that physiological differences exist. Therefore, the general application of our results is dependent on the lifestyles and ethnicity of various populations.

## 5. Conclusions

We found that DED in Japanese patients with type 2 diabetes was positively and strongly associated with BMI, and the prevalence of obesity could potentially be used for dietary assessment and education of people with type 2 diabetes. It was also revealed that DED is much more potently associated with obesity and BMI than established indicators such as amounts of energy or macronutrients that have been traditionally used for dietary evaluation for weight control. Further investigations are necessary to confirm the usefulness of DED for weight management in people with diabetes mellitus through longitudinal and interventional studies.

## Figures and Tables

**Table 1:** Characteristics of participants, nutritional factors, and food groups according to quintiles of energy density.

	Energy Density (kcal/g)					
	Q1	Q2	Q3	Q4	Q5	
	<1.34	1.35–1.44	1.45–1.55	1.56–1.70	≥1.71	*p* for Trend
*n*	317	307	314	354	323	
Energy density	1.23 ± 0.09	1.39 ± 0.03	1.50 ± 0.03	1.62 ± 0.04	1.86 ± 0.16	<0.001
Women (%)	177 (55.8)	135 (44.0)	112 (35.7)	110 (31.1)	76 (23.5)	<0.001
Age (years)	67.4 ± 10.1	63.8 ± 11.2	64 ± 12.1	60.6 ± 12.1	54.4 ± 11.3	<0.001
BMI (kg/m^2^)	24.2 ± 3.9	25.4 ± 4.5	25.8 ± 4.6	26.3 ± 4.7	27.6 ± 5.0	<0.001
Duration (years)	13.1 ± 9.1	11.9 ± 7.6	11.6 ± 7.8	10.8 ± 7.2	9.4 ± 7.6	<0.001
Systolic Blood Pressure (mmHg)	127.3 ± 15.3	126.9 ± 14.9	127.5 ± 14.5	127.4 ± 15.4	125.1 ± 14.8	0.177
HbA1c (%) (mmol/mol)	7.2 (55) ± 1.1	7.2 (55) ± 1.1	7.2 (55) ± 1.0	7.3 (56) ± 1.2	7.5 (56) ± 1.3	0.003
Non-HDL Cholesterol (mmol/L)	3.4 ± 0.9	3.5 ± 0.9	3.5 ± 0.9	3.5 ± 1.0	3.6 ± 0.9	0.072
Treated by Insulin (%)	102 (32.2)	109 (35.5)	87 (27.7)	90 (25.4)	73 (22.6)	0.002
OHA without Insulin (%)	171 (53.9)	160 (52.1)	180 (57.3)	204 (57.6)	191 (59.1)	0.366
Physical Activity (METs·h/w)	31.1 ± 39.2	25.5 ± 35.3	28.6 ± 39.7	33.5 ± 45.8	31.6 ± 49.2	0.224
Current Smoker (%)	33 (10.4)	46 (15.0)	47 (15.0)	85 (24.0)	101 (31.3)	<0.001
Energy (kcal)	1618 ± 411	1757 ± 409	1798 ± 394	1818 ± 514	1855 ± 571	<0.001
Volume of food (g)	1209 ± 289	1171 ± 272	1116 ± 237	1039 ± 303	918 ± 293	<0.001
Fat (g)	50 ± 14.6	56.9 ± 18.3	59 ± 17.5	60.7 ± 21.1	66.2 ± 27.5	<0.001
(% Energy)	28.0 ± 5.4	28.9 ± 5.2	29.3 ± 4.9	29.9 ± 5.4	31.6 ± 5.8	<0.001
Carbohydrate (g)	216 ± 63.1	228.6 ± 55.7	235.5 ± 53.9	236.2 ± 72.8	233.2 ± 69.7	<0.001
(% Energy)	55.9 ± 6.9	55.6 ± 6.4	55.9 ± 6.2	55.8 ± 6.7	55.0 ± 6.9	0.196
Protein (g)	65.0 ± 17.7	68.1 ± 18.6	66.6 ± 16.8	65.0 ± 20.8	62.5 ± 25.2	<0.001
(% Energy)	16.1 ± 2.2	15.5 ± 2.1	14.8 ± 2.0	14.3 ± 1.9	13.4 ± 2.0	<0.001
Total Dietary Fiber (g)	15.8 ± 4	13.9 ± 3.7	12.7 ± 3.5	11.5 ± 4.1	9.8 ± 3.4	<0.001
Soluble Dietary Fiber (g)	3.6 ± 1.0	3.2 ± 1.0	2.9 ± 0.9	2.7 ± 1.1	2.4 ± 0.9	<0.001
Insoluble Dietary Fiber (g)	11.4 ± 2.9	10.0 ± 2.7	9.2 ± 2.5	8.3 ± 2.9	7.0 ± 2.5	<0.001
Salt (g)	8.5 ± 3.3	8.9 ± 3.3	9.0 ± 3.0	8.6 ± 3.4	8.0 ± 3.3	0.018
Steamed white rice (g)	229.4 ± 123.8	258.6 ± 121.4	274.4 ± 109.8	268.9 ± 140.7	247.2 ± 124.8	0.036
Bread (g)	28.9 ± 28.6	33.3 ± 32.4	33.3 ± 30.3	36.9 ± 37.6	44.7 ± 40.5	<0.001
Noodles (g)	61.6 ± 56.1	65.5 ± 60.0	66.7 ± 56.2	66.2 ± 55.6	65.3 ± 54.7	0.418
Potato (g)	38.9 ± 34.2	30.4 ± 28.0	30.5 ± 24.6	24.9 ± 24.7	16.8 ± 17.2	<0.001
Green and yellow vegetables (g)	123.5 ± 48.1	98.2 ± 40.3	78.9 ± 37.3	62.2 ± 35.0	41.7 ± 29.9	<0.001
Other vegetables (g)	230.2 ± 77.5	186 ± 68.7	149.9 ± 66.0	119.6 ± 63.1	79.3 ± 48.0	<0.001
Seaweed (g)	6.6 ± 4.6	5.3 ± 3.4	4.8 ± 3.8	4.0 ± 3.3	3.0 ± 3.1	<0.001
Beans (g)	80.6 ± 48.3	66.8 ± 42.1	63.4 ± 40.9	53.1 ± 38.0	41.5 ± 33.5	<0.001
Seafood and fish (g)	83.4 ± 46.2	85.3 ± 46.5	74.7 ± 42.1	69.2 ± 42.4	52.5 ± 54.7	<0.001
Meat (g)	53.1 ± 33.5	72.2 ± 45.5	78.5 ± 47.6	83.2 ± 52.0	95.1 ± 67.7	<0.001
Eggs (g)	26.5 ± 17.3	27.4 ± 19.4	28.0 ± 18.8	27.3 ± 20.7	26.8 ± 18.2	0.876
Milk (g)	94.7 ± 104.6	90.3 ± 95.3	83.0 ± 77.8	73.4 ± 75.2	72.0 ± 96.2	<0.001
Dairy products (g)	52.9 ± 38.3	56.9 ± 44.5	48.5 ± 37.8	45.7 ± 37.9	42.2 ± 38.0	<0.001
Fruits (g)	124.3 ± 74.9	100.5 ± 70.5	87.0 ± 66.3	71.1 ± 64.0	35.6 ± 41.1	<0.001
Confectionery (g)	28.0 ± 24.5	38.8 ± 33.1	49.4 ± 38.6	60.6 ± 43.1	81.7 ± 56.2	<0.001
Alcohol (g)	75.3 ± 126.4	83.7 ± 131.3	76.3 ± 135.8	85.8 ± 132.7	94.7 ± 156.2	0.089
Other beverages (g)	16.5 ± 71.9	23.5 ± 62.6	36.4 ± 78.6	55.9 ± 115.6	73.7 ± 126.3	<0.001
Sugar (g)	8.4 ± 5.3	8.1 ± 5.7	7.9 ± 5.4	6.6 ± 5.0	5.2 ± 5.2	<0.001
Seeds (g)	3.3 ± 5.3	2.8 ± 4.8	2.6 ± 4.8	3.1 ± 5.2	3.0 ± 7.1	0.946
Oil and fat (g)	9.9 ± 7.2	11.9 ± 7.9	13.1 ± 8.8	12.4 ± 7.4	13.4 ± 7.3	<0.001
Seasoning and spices (g)	19.7 ± 10.8	22.6 ± 12.5	24.3 ± 13.2	24 ± 13.7	23.4 ± 13.2	<0.001

Data are means ± SD, or *n* (%). *p* trend values are from linear regression for continuous variables and χ^2^ test for categorical variables. There are significant trends in differences in energy intake among quintiles. Higher intakes of energy and fat are shown from the first to the fifth quintiles of DED (approximately 14% and 32% higher, respectively). In contrast, a significantly (approximately 38%) lower intake of total dietary fiber is shown from the first to the fifth quintiles. Significant trends are also observed for the intake of soluble and insoluble dietary fiber (approximately 33% and 39% lower, respectively).

**Table 2:** Odds ratios (ORs) for obesity (BMI ≥ 25) with 95% CIs for energy density (DED), intake of energy, and macronutrients according to quintiles.

		Range	Case/*n*	ORs (95% CI)	*p*	*p* for Trend
DED (kcal/g)	Q1	–1.34	118/316	Ref.		<0.001
	Q2	1.35–1.44	140/325	1.58 (1.13–2.20)	0.007	
	Q3	1.45–1.55	175/315	1.66 (1.19–2.32)	0.003	
	Q4	1.56–1.70	186/319	2.24 (1.61–3.12)	<0.001	
	Q5	1.71–	239/340	2.99 (2.01–3.12)	<0.001	
Energy intake (kcal)	Q1	–1416.4	172/323	Ref.		0.224
	Q2	1416.5–1624.7	163/323	0.88 (0.63–1.21)	0.418	
	Q3	1624.8–1829.4	167/323	1.01 (0.73–1.39)	0.963	
	Q4	1829.5–2081.1	173/322	1.03 (0.74–1.43)	0.863	
	Q5	2081.2–	183/324	1.17 (0.84–1.63)	0.361	
Fat (g)	Q1	–42.1	163/322	Ref.		0.173
	Q2	42.2–51.6	164/320	1.02 (0.74–1.41)	0.887	
	Q3	51.7–60.4	161/327	0.89 (0.65–1.23)	0.484	
	Q4	60.6–72.9	175/323	1.11 (0.80–1.54)	0.529	
	Q5	73.0–	195/323	1.25 (0.90–1.74)	0.187	
Carbohydrate (g)	Q1	–186.4	172/323	Ref.		0.041
	Q2	186.5–214.6	157/320	0.89 (0.64–1.23)	0.483	
	Q3	214.7–238.3	170/326	1.14 (0.82–1.58)	0.427	
	Q4	238.4–271.5	185/323	1.30 (0.94–1.81)	0.114	
	Q5	271.7–	174/323	1.22 (0.88–1.70)	0.240	
Protein (g)	Q1	–49.4	185/319	Ref.		0.595
	Q2	49.5–59.6	174/327	0.87 (0.63–1.20)	0.389	
	Q3	59.7–68.1	158/318	0.78 (0.56–1.08)	0.137	
	Q4	68.2–79.2	167/327	0.82 (0.59–1.14)	0.243	
	Q5	79.3–	174/324	0.93 (0.67–1.29)	0.654	

**Table 3:** β coefficient of BMI with per 1 SD increment according to DED, intake of energy, and macronutrients.

		β (95% CIs)	*p* Value
DED			
	age, sex adjusted	0.64 (0.41–0.88)	<0.001
	multivariate adjusted	0.65 (0.41–0.88)	<0.001
Energy Intake			
	age, sex adjusted	0.22 (0.00–0.44)	0.051
	multivariate adjusted	0.29 (0.07–0.51)	0.011
Fat			
	age, sex adjusted	0.35 (0.13–0.57)	0.002
	multivariate adjusted	0.38 (0.16–0.60)	0.001
Carbohydrate			
	age, sex adjusted	0.19 (−0.03–0.40)	0.090
	multivariate adjusted	0.21 (−0.01–0.43)	0.059
Protein			
	age, sex adjusted	0.05 (−0.16–0.27)	0.624
	multivariate adjusted	0.08 (−1.35–0.30)	0.462

β coefficients are interpreted as the amount of change in BMI associated with 1-SD increasing change in each variable. Multivariate adjusted models: age, sex, diabetes duration, treated by insulin, treated by OHA, physical activity, smoking status, alcohol intake.

**Table 4:** ORs for obesity (BMI ≥ 25) with 95% CIs for per 1 SD increment of DED, intake of energy, macronutrients.

		ORs (95% CIs)	*p* Value
DED			
	age, sex adjusted	1.45 (1.29–1.64)	<0.001
	multivariate adjusted	1.48 (1.31–1.67)	<0.001
Energy Intake			
	age, sex adjusted	1.09 (0.99–1.21)	0.089
	multivariate adjusted	1.14 (1.03–1.27)	0.015
Fat			
	age, sex adjusted	1.13 (1.02–1.26)	0.021
	multivariate adjusted	1.16 (1.04–1.29)	0.007
Carbohydrate			
	age, sex adjusted	1.08 (0.98–1.20)	0.127
	multivariate adjusted	1.12 (1.00–1.24)	0.042
Protein			
	age, sex adjusted	1.00 (0.91–1.11)	0.958
	multivariate adjusted	1.03 (0.92–1.14)	0.636

**Table 5:** Standardized β coefficients from multiple linear regression models for food groups intake.

Food Groups	Standardized β	*p* Value
Confectioneries	0.19	<0.001
Bread	0.10	<0.001
Oil and fat	0.09	<0.001
Meat	0.06	<0.001
Seeds	0.05	<0.001
Sugar	0.02	0.147
Seasoning and spices	−0.01	0.507
Seaweed	−0.02	0.079
Eggs	−0.06	<0.001
Noodles	−0.07	<0.001
Beans	−0.09	<0.001
Dairy products	−0.09	<0.001
Potato	−0.10	<0.001
Seafood and fish	−0.13	<0.001
Steamed white rice	−0.14	<0.001
Green and yellow vegetables	−0.25	<0.001
Fruits	−0.31	<0.001
Other vegetables	−0.43	<0.001

β coefficients are standardized and are interpreted as the amount of 1 kcal/g change in DED associated with a gram change in food groups variable. Other adjusted factors: age, sex, energy intake, physical activity, smoking status, alcohol intake.

## Data Availability

We are unable to provide an anonymized data set containing our underlying data used to create the figures and tables because these data are private property of the JDDM Study Group. Making these data available to the general public will result in loss of ownership of the data by the JDDM Study Group.
